# Circular RNA BCRC-3 suppresses bladder cancer proliferation through miR-182-5p/p27 axis

**DOI:** 10.1186/s12943-018-0892-z

**Published:** 2018-10-03

**Authors:** Fei Xie, Yawei Li, Miao Wang, Chao Huang, Dan Tao, Fuxin Zheng, Hui Zhang, Fuqing Zeng, Xingyuan Xiao, Guosong Jiang

**Affiliations:** 1 Department of Urology, Union Hospital, Tongji Medical College, Huazhong University of Science and Technology, Wuhan, 430022 China; 2 Department of Urology, The First Affiliated Hospital of Wannan Medical College, Wuhu, 241001 China; 3 Department of Oncology, The Fifth Hospital of Wuhan, Wuhan, 430050 China

**Keywords:** CircRNAs, Bladder cancer, BCRC-3, miR-182-5p, p27, 3’UTR, MJ

## Abstract

**Background:**

Circular RNAs (circRNAs) are a new member of noncoding RNAs (ncRNAs) that have recently been described as key regulators of gene expression. Our previous study had identified the negative correlation between circHIPK3 and bladder cancer grade, invasion, as well as lymph node metastasis. However, the roles of circRNAs in cellular proliferation in bladder cancer remain largely unknown.

**Methods:**

We had analyzed circRNA high-throughout sequencing from human tissues and determined bladder cancer related circRNA-3 (BCRC-3, GenBank: KU921434.1) as a new candidate circRNA derived from PSMD1 gene. The expression levels of circRNAs, mRNAs and miRNAs in human tissues and cells were detected by quantitative real-time PCR (qRT-PCR). The effects of BCRC-3 on cancer cells were explored by transfecting with plasmids in vitro and in vivo. RNA pull down assay, luciferase reporter assay and fluorescence in situ hybridization were applied to verify the interaction between BCRC-3 and microRNAs. Anticancer effects of methyl jasmonate (MJ) were measured by flow cytometry assay, western blot and qRT-PCR.

**Results:**

BCRC-3 was lowly expressed in bladder cancer tissues and cell lines. Proliferation of BC cells was suppressed by ectopic expression of BCRC-3 in vitro and in vivo. Mechanistically, overexpression of BCRC-3 induced the expression of cyclin-dependent kinase inhibitor 1B (p27). Importantly, BCRC-3 could directly interact with miR-182-5p, and subsequently act as a miRNA sponge to promote the miR-182-5p-targeted 3’UTR activity of p27. Furthermore, MJ significantly increased the expression of BCRC-3, resulting in an obvious up-regulation of p27.

**Conclusions:**

BCRC-3 functions as a tumor inhibitor to suppress BC cell proliferation through miR-182-5p/p27 axis, which would be a novel target for BC therapy.

**Electronic supplementary material:**

The online version of this article (10.1186/s12943-018-0892-z) contains supplementary material, which is available to authorized users.

## Background

Bladder cancer (BC) is the number one malignancy of urinary system with an estimated over 79,030 deaths predicted in 2017 in the United State [1].The high rate of recurrence and distant metastasis of BC created a huge economic burden in EU [2]. New technology like the blue-light cystoscopy has been proved to improve the detection of BC, especially flat lesions [3]. However, the researches on early diagnostic assessment and specific markers for BC are still deficient [4]. The guideline provides recommended treatment based on the grade and stage of BC [5, 6], ranging from radical cystectomy to systemic chemotherapy. Nevertheless, the overall therapeutic effects of BC are limited and the five-year survival rate keeps at a low level [7, 8]. Thus, further exploration of genetic regulatory networks involved in BC progression and development of precise strategies are worthy and important.

Circular RNAs (circRNAs), a new member of noncoding RNAs (ncRNAs), have attracted great attentions for their closed continuous loop structure and potential value in clinical work [9, 10]. CircRNAs were found in cells in the 1970s by electron microscope [11, 12]. The development of the high-throughout sequencing and computational approaches have identified more than 30,000 circRNAs and proved that they are endogenous, abundant and conserved in mammalian cells [13–15]. Importantly, studies have demonstrated that circRNAs are closely related to neurological disorders, atherosclerotic vascular disease risk, carcinomas and so on [16–18]. Some circRNAs contain miRNA response elements (MREs) and function as competing endogenous RNAs (ceRNAs) to interact with miRNAs and regulate the expression of target mRNAs. The studies of CiRS-7 provided the solid evidence for this notion [16, 19]. CiRS-7 has more than 70 miR-7 binding sites and thus acts as effective miR-7 suppressor to regulate the expression of miR-7 target mRNAs. Recently, circBIRC6 has been found to directly interact with miR-34a and miR-145 to modulate target genes that maintain pluripotency [20], and it is reported that CircMTO1 suppresses human hepatocellular carcinoma progression by acting as the sponge of oncogenic miR-9 to promote p21 expression [21]. In general, the circRNA-miRNA-mRNA axis may function as an extensive regulatory network in progression of some diseases.

In our previous study, we found 6154 distinct circRNAs from human BC and normal bladder tissues by performing RNA-seq, and identified circHIPK3 as a tumor suppressor in BC [22]. CircHIPK3 inhibits migration, invasion, and angiogenesis of BC cells via acting as “miRNA sponge” for miR-558. In this research, we mainly focus on the impacts of circRNAs on BC cell proliferation and characterize a circRNA derived from PSMD1 gene (bladder cancer related circRNA-3, BCRC-3). BCRC-3 is significantly down-regulated in BC tissues and effectively inhibits the proliferation of BC cells. Importantly, our study reveals that BCRC-3 could bind to oncogenic miR-182-5p to promote p27 expression and therefore inhibit BC progression. Collectively, BCRC-3 may serve as a novel promising target for BC treatment.

## Methods

### Patient tissue specimens and cell lines

A total of 47 BC tissues and their adjacent normal bladder tissues were obtained from patients who underwent radical cystectomy for urothelial carcinomas of bladder at the Department of Urology of Union Hospital affiliated of Tongji Medical College between 2015 and 2017. We have received the approval from the Institutional Review Board of Tongji Medical College of Huazhong University of Science and Technology before we collected the samples. All specimens were classified according to the 2004 World Health Organization Consensus Classification and Staging System for bladder neoplasms. Clinicopathological characteristics of patients are shown in Additional file 1: Table S1. The human BC cell line EJ and normal human urothelial cells SV-HUC-1 were obtained from American Type Culture Collection (ATCC, Manassas, VA, USA). The human BC cell line T24 T was provided by Dr. Dan Theodorescu (Departments of Urology, University of Virginia, Charlottesville, VA) as described in our previous studies [23]. All the cell lines were cultured in RPMI-1640 medium (Gibco, Grand Island, NY, USA) supplemented with 10% fetal bovine serum (Gibco, Australia origin) and 1% penicillin/streptomycin (Gibco) in the recommended media at 37 °C supplied with 5% CO_2_.

### RNA extraction, RNase R treatment and PCR assays

Total RNA was isolated from tissues and cell lines with RNeasy Mini Kit (QIAGEN, Germany) according to the manufacturer’s instructions. RNase R treatment was processed at 37 °C with 3 U/mg of RNase R (Epicenter, WI, USA) for 15 min. Complementary DNA was synthesized using random primers and the reverse transcription kit PrimeScript RT Master Mix (Takara, Dalian, China) or commercial miRNA reverse transcription PCR kit (RiboBio, Guangzhou, China). Genomic DNA (gDNA) was isolated with QIAamp DNA Mini Kit (QIAGEN, Germany). Quantitative real-time PCR (qRT-PCR) analysis was carried out using the SYBR Premix Ex TaqTM kit (Takara). The differences of circRNA and miRNA were normalized to the levels of GAPDH or U6. All data were analyzed via the StepOnePlus Real-Time PCR System (Applied Biosystems, Carlsbad, CA, USA). Bulge-Loop miRNAs qPCR primers were obtained from RiboBio. The details of primers are listed in Additional file 1: Table S2.

### Plasmids construction and stable transfection

The human BCRC-3 and p27 3’UTR cDNA was synthesized by TSINGKE (Wuhan, China). BCRC-3 was cloned into pCD-ciR vector (Geenseed Biotech Co, Guangzhou, China) which contained a front circular frame and a back circular frame. P27 3’UTR was cloned into pMIR-REPORT vector. The plasmids of p27 3’UTR T1, T2, T3 and mutant luciferase reporters were synthesized using Trelief™ SoSoo Cloning Kit (TSINGKE, Beijing, China). The p27 promoter luciferase reporter vector was constructed and used as our previous study described [24]. MiR-182-5p mimics and its control were purchased from RiboBio (Guangzhou, China). SiRNA aimed at BCRC-3 was synthesized by Gene-Pharma (Shanghai, China). ShRNAs targeting p27 were designed and synthesized by Genechem (Shanghai, China). Lipofectamine 2000 (Life Technologies) was used for plasmid transfection following the manufacturer’s instructions. The cells transfected BCRC-3 were screened with G418 (Life Technologies) for 4–6 weeks.

### RNA –FISH

Cy3-labeled BCRC-3 and Dig-labeled locked nucleic acid miR-182-5p probes were purchased from RiboBio (Guangzhou, China). The images were obtained using Fluorescent in Situ Hybridization kit (RiboBio) following the manufacturer’s instructions. All data were analyzed via Nikon A1Si Laser Scanning Confocal Microscope (Nikon Instruments Inc., Japan).

### Flow cytometry assay for the cell cycle

EJ and T24 T cells transfected with the plasmids or treated with MJ were harvested and stained with propidium iodide buffer (BD Pharmingen) for cell cycle analysis. The results were analyzed by the ModFit LT software.

### Colony formation assay

EJ and T24 T cells transfected with the plasmids were cultured in 6-wells plates at density of 800–1000 cells per well. Plates were incubated at 37 °C in 5% CO_2_ for 2–3 weeks, and the colonies with more than 50 cells were scored. The cell colonies were immobilized with 4% paraformaldehyde and dyed by coomassie brilliant blue.

### 5-Ethynyl-20-deoxyuridine (EdU) assay

Cell-Light EdU DNA Cell Proliferation Kit (C10310–1, RiboBio Guangzhou, China) was used to assess cell proliferation viability following the manufacturer’s protocol. All images were obtained with an Olympus FSX100 microscope (Olympus, Tokyo, Japan). The ratio of EdU-stained cells to Hoechst-stained cells was calculated to evaluate the cell proliferation as described [25].

### Tumor xenografts

We chose 4-week-old female BALB/c nude mice for tumor xenografts experiments. T24 T cells stably transfected with BCRC-3 plasmids or control vector were subcutaneously injected into the upper back of the nude mice (3 × 10^6^, 200 μl). Mice were sacrificed and detected for tumor weight, gene expression after one month. All procedures were approved by the Animal Care Committee of Tongji Medical College.

### Western blotting analysis

The proteins were extracted in RIPA lysis buffer (Thermo Scientific) and determined using BCA Protein assay kit (Beyotime). After separated by electrophoresis and transferred onto PVDF membranes, total proteins were incubated with primary antibodies overnight. The membranes were blocked for 1 h in the specific HRP-conjugated secondary antibodies at room temperature. All images were obtained by using BioSpectrum 600 Imaging System (UVP, CA, USA). Antibodies against CDK2 (Cat No: 10122–1-AP), CDK4 (Cat No: 11026–1-AP), CDK6 (Cat. No: 14052–1-AP), cyclin D1 (Cat. No: 60186–1-Ig), Cyclin E (Cat. No: 11554–1-AP), p21 (Cat. No: 10355–1-AP), p27 (Cat. No: 25614–1-AP), β-actin (Cat. No: 60008–1-Ig), HRP-conjugated secondary goat anti-mouse (Cat. No: SA00001–1) and goat anti-rabbit (Cat. No: SA00001–2) were purchased from Proteintech Group (Chicago, USA).

### Pull-down assay with biotinylated BCRC-3 probe

Biotinylated-BCRC-3 probe was synthesized by RiboBio (Guangzhou, China). The sequence of the probe was just complemented to the back-spliced junction of BCRC-3 (listed in Additional file 1: Table S2). Pull-down assay was carried out as described in our previous study [22]. The RNA complexes combining on the beads were finally extracted with RNeasy Mini Kit (QIAGEN, China) for further research.

### Pull-down assay with biotinylated miRNA

Biotinylated miRNA mimics or their mutants were synthesized by RiboBio (Guangzhou, China). The sequences are listed in Additional file 2: Figure S4b. The pull-down assay with biotinylated miRNA was performed as described in our previous study [22]. The bound RNAs were purified using RNeasy Mini Kit (QIAGEN) for further analysis.

### Immunohistochemistry analysis

The primary antibody used to detect p27 was purchased from Proteintech Group (Chicago, USA). The immunostaining images were captured using Olympus FSX100 microscope (Olympus, Japan). Protein expression levels were analyzed by calculating the integrated optical density as described [26].

### Luciferase reporter assays

The p27 3’UTR or promoter reporters were transiently transfected along with Renilla control plasmid, and the BC cells were co-tranfected with BCRC-3, miR-182-5p mimics, or co-treated with MJ, respectively. The luciferase activities were measured following dual luciferase reporter assay detection kit (Promega, WI, USA) as described [27] after 24 h.

### Statistical analysis

All data were indicated as means ± standard error of the mean (SEM) processed by GraphPad Prism 5.0 (La Jolla, USA). Student’s *t*-test or chi-square (*P* < 0.05) was used to evaluate the Group difference.

## Results

### BCRC-3 is down-expressed in BC tissues and cell lines, and is predominantly localized in the cell cytoplasm

**Fig. 1:**
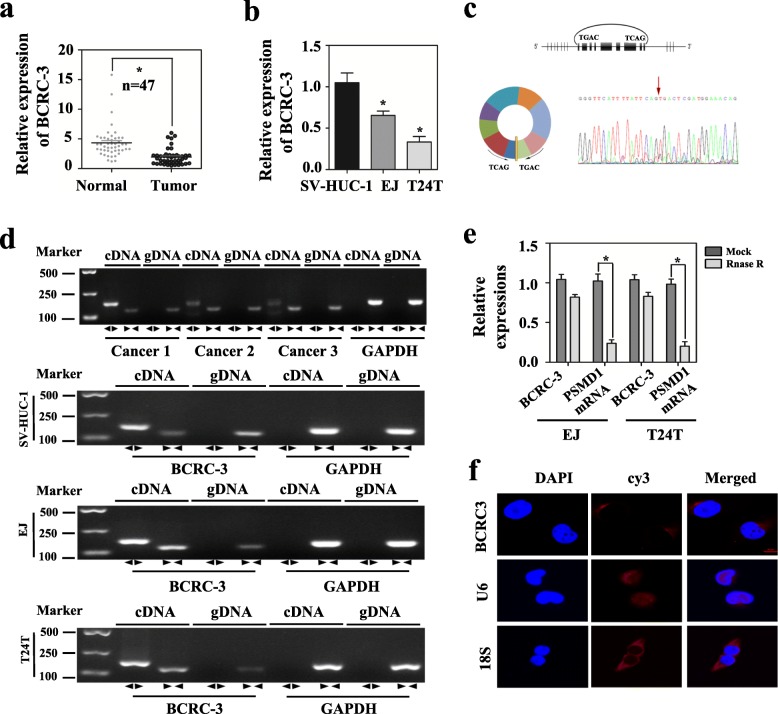
The expression of BCRC-3 in cell lines and tissues, and the subcellular location of BCRC-3. **a** qRT-PCR assay with divergent primers indicated the low expression of BCRC-3 in 47 pairs of human BC compared with their adjacent normal tissues. **b** The expression of BCRC-3 in SV-HUC-1, EJ and T24 T cell lines were measured by qRT-PCR. **c** Schematic diagram demonstrated that nine exons derived from PSMD1 constituted BCRC-3. The existence of BCRC-3 was proved by RT–PCR and its back splicing junction was verified by Sanger sequencing. Red arrow indicated the special splicing junction of BCRC-3. **d** RT-PCR assay with divergent or convergent primers indicating the existence of BCRC-3 in SV-HUC-1, EJ, T24 T cell lines and three BC tissues. GAPDH was used as negative control. **e** qRT-PCR analysis of the expression of BCRC-3 after RNase R treatmet in EJ or T24 T cells. **f** RNA-FISH indicated the location of BCRC-3 in T24 T cells. U6 was used as negative control and 18S was used as positive control. Nuclei were stained blue by DAPI. BCRC-3, U6 and 18S were stained red with cy3 (scale bar, 10 μm). (Data are mean ± SEM of three experiments. Student’s *t*-test analyzed the difference in **a**-**b**, **e**. * *P* < 0.01 vs. normal bladder tissues, SV-HUC-1, or mock)

Our previous studies had characterized circular RNA transcripts using RNA-seq analysis of ribosomal RNA-depleted total RNA from three paired normal and cancerous bladder tissues [22]. Based on this data, we further verified a decreased expression of circRNA BCRC-3 in BC tissues and adjacent normal tissues (*n* = 47) as well as in BC cells by using qRT-PCR analysis, and had submitted the sequence data of this circRNA to GenBank (KU921434.1). Consistent with the RNA-seq results, BCRC-3 was significantly down-regulated in BC tissues (Fig. 1a). Nevertheless, the expression of BCRC-3 had no relationship with BC grade and pathological stage (Additional file 1: Table S1). Besides, BCRC-3 was also expressed at low level in EJ and T24 T BC cell lines, in comparison to human immortalized uroepithelium cells SV-HUC-1 (Fig. 1b).

The genomic structure indicates that BCRC-3 consists of nine exons (1,002 bp) from the PSMD1 gene (Fig. 1c). This circular product was amplified by RT–PCR with divergent primers and confirmed by Sanger sequencing (Fig. 1c). To rule out the possibilities of trans-splicing or genomic rearrangements, two steps were taken to prove the existence of the head-to-tail splicing. Firstly, we designed convergent primers and divergent primers to amplify linear and circular RNA based on cDNA and genomic DNA (gDNA) from three BC tissues and SV-HUC-1, EJ, T24 T cell lines by RT-PCR. As shown in Fig. 1d, BCRC-3 could only be amplified by primers in cDNA, but not in gDNA. Secondly, RNase R was used to pretreat the RNAs, and it showed that the circular form was resistant to RNase R, while the linear RNA was significantly reduced after RNase R treatment. The information about the primers was listed in Additional file 1: Table S2.

Then, we performed RNA fluorescence in situ hybridization (FISH) assay to identify the subcellular localization of BCRC-3. Cy3-labeled probe specific for BCRC-3 was used for RNA-FISH. The images indicated that BCRC-3 was mainly localized in a punctate pattern in the cytoplasm (Fig. 1f). These results demonstrated that BCRC-3 was relatively low-expressed in BC tissues and cell lines, and was predominantly localized in cell cytoplasm.

### Overexpression of BCRC-3 suppresses cell growth in BC cells in vitro and inhibits BC tumor growth in vivo

**Fig. 2:**
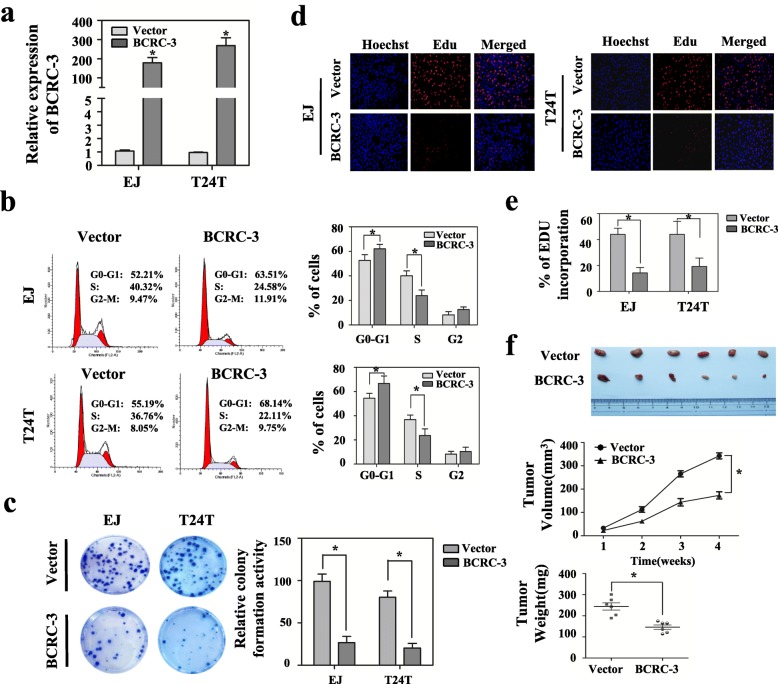
Enforced expression of BCRC-3 inhibits cell proliferation in vitro and impairs tumor growth in vivo. **a** qRT-PCR analysis verified the effective overexpression of BCRC-3 after transfection of the vector for 48 h in EJ or T24 T cells. **b** Cell cycle distributions in BCRC-3 overexpression cells were presented by flow cytometry. **c** Cloning formation assay of EJ or T24 T cells transfected with BCRC-3 overexpression vectors. Colonies with more than 50 cells were counted. **d** & **e** Observation of DNA synthesis of EJ or T24 T cells transfected with BCRC-3 overexpression vectors by EdU assay. **f** T24 T cells with stable overexpression of BCRC-3 or vector were injected in the flanks (5 × 10^6^cells per mouse, *n* = 6 for each group). Tumors collected from mice were measured and weighed after one month of hypodermic injection. (Data are mean ± SEM of three experiments. Student’s *t*-test analyzed the difference in **a**-**c**, **e**-**f**. * *P* < 0.01 vs. vector)

To investigate the function of BCRC-3 in BC cells, BCRC-3 overexpression plasmid was stably transfected into EJ or T24 T cells with G418 antibiotic selection. The results from qRT-PCR indicated that BCRC-3 was up-regulated over about 150 folds and 200 folds in EJ and T24 T stable transfectants, respectively (Fig. 2a). The cell cycle assay suggested that BCRC-3 overexpression induced cell cycle arrest at G0/G1 phase in EJ and T24 T cells (Fig. 2b). We then evaluated the growth capability of stable transfectants using plate clone formation assay. As shown in Fig. 2c, overexpression of BCRC-3 led to significant inhibition of cell proliferation. Consistently, the EdU assay demonstrated that ectopic expression of BCRC-3 suppressed proliferation of EJ and T24 T cells (Fig. 2d and e). On the other hand, knockdown of BCRC-3 promoted proliferation and stimulated cell cycle progression of BC cells (Additional file 2: Figure S1a-d).

To further test the function of BCRC-3 in vivo, T24 T cells stably transfected with BCRC-3 or control vector were respectively injected into nude mice. The decreased growth rate and tumor weight of xenograft tumors were established in BCRC-3 transfectants compared with vector group (Fig. 2f). Collectively, these results suggested that BCRC-3 played a role as tumor suppressor through inhibiting proliferation and cell cycle progression in BC cells.

### BCRC-3 inhibit BC cell proliferation through increasing the expression of p27

**Fig. 3:**
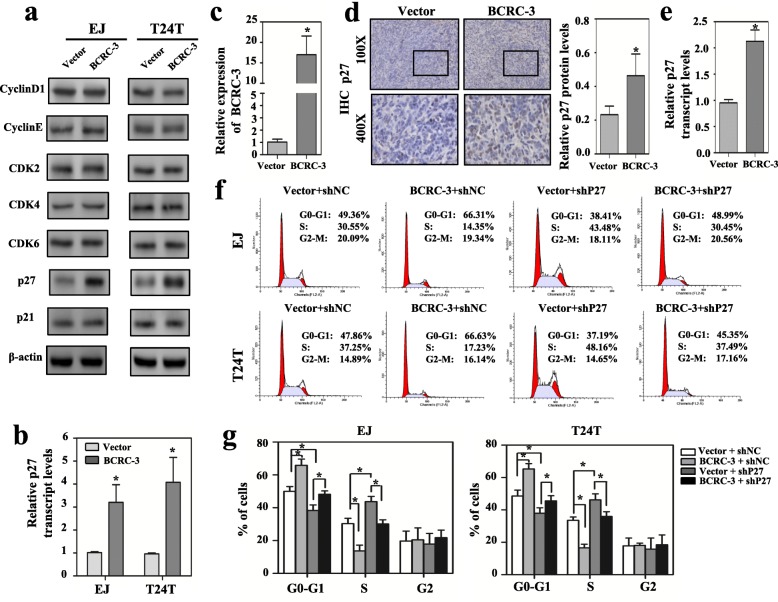
Overexpression of BCRC-3 up-regulates p27 expression. **a** Western blot analysis of the expression levels of cell cycle related proteins (p27, CDK2, CDK4, CDK6, CyclinD1, CyclinE, p21) after transfection with BCRC-3 overexpression vectors in EJ or T24 T cell lines. **b** The p27 mRNA expression was detected by qRT-PCR. **c** qRT-PCR analysis of the expression of BCRC-3 in xenograft tumors from mice (Fig. 2f). **d** P27 expression levels in xenograft tumors were determined by immunohistochemistry (IHC) staining and analyzed by calculating the integrated optical density per stained area (IOD/area). **e** The p27 mRNA expression of xenograft tumors was detected by qRT-PCR. Data are means ± SEM of 6 mice from each group. **f** & **g** Cell cycle distributions in BCRC-3 overexpression vector and shP27 co-transfected cells were presented by flow cytometry. (The results are mean ± SEM of three experiments. Student’s *t*-test compared the difference in **b**-**e** and **g**. * *P* < 0.01 vs. vector, vector + shNC, or vector + shP27)

To investigate the underlying mechanisms of BCRC-3 that suppressed cell proliferation, a series of key proteins related with cell cycle were measured by western blot assay, including cyclin D1, cyclin E, CDK2, CDK4, CDK6, p27 and p21. As shown in Fig. 3a, p27 was the only up-regulated protein in BCRC-3 overexpression stable transfectants. The results from qRT-PCR suggested that BCRC-3 overexpression also led to the up-regulation of p27 mRNA expression (Fig. 3b). In addition, the expression of p27 was significantly decreased in BCRC-3 knockdown BC cells (Additional file 2: Figure S1e & f). To explore the effect of BCRC-3 on p27 expression in vivo, we detected protein and mRNA levels of xenografted tumor. The results indicated that BCRC-3-induced inhibition of tumor growth (Fig. 3c) was accompanied by p27 up-regulation (Fig. 3d and e), which was consistent with the in vitro data.

We then designed shRNAs targeting p27 (shP27) to further text whether p27 was involved in BCRC-3-induced cell cycle arrest. The shP27–3 was ultimately selected for further studies because of its better knockdown effects (Additional file 2: Figure S2a). It showed that knockdown of p27 promoted cell cycle progression in BC cells (Fig. 3f and g). Moreover, BCRC-3-induced cell cycle arrest was partially reversed upon knockdown of p27 (Fig. 3f and 3g). The verification of BCRC-3 overexpression in the co-tranfected cells was shown in Additional file 2: Figure S2b. Therefore, we further confirmed that BCRC-3 could suppress cell proliferation via increasing the expression of p27.

### BCRC-3 promotes p27 expression by interacting with miR-182-5p in BC cells

**Fig. 4:**
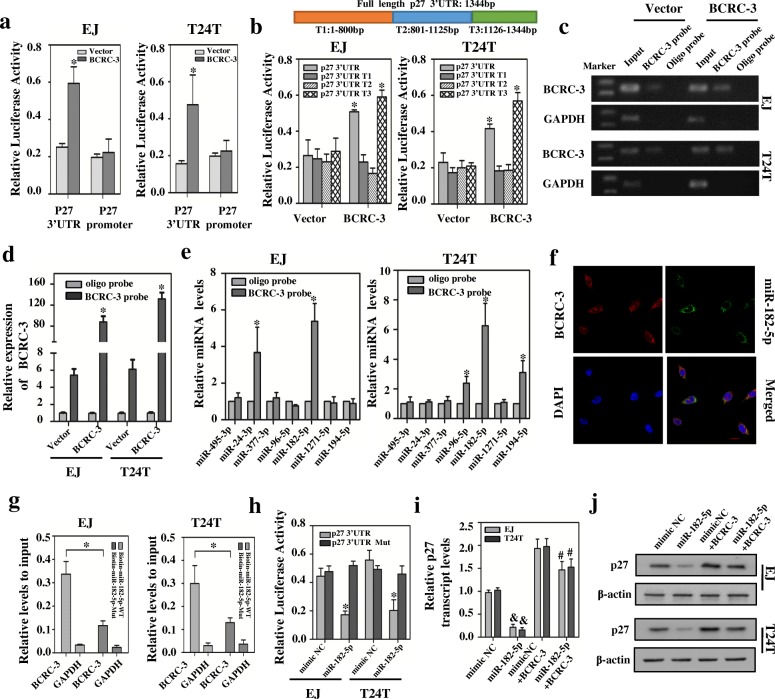
BCRC-3 sponges miR-182-5p, and overexpression of BCRC-3 reverses the down-regulation effect of miR-182-5p on p27. **a** The luciferase activity of p27 3’UTR and promoter after transfection with BCRC-3 overexpression vectors in EJ or T24 T cell lines. **b** The relative luciferase activities of p27 3’UTR and truncates in EJ or T24 T cells transfected with BCRC-3 overexpression vector. Schematic Sequence of p27 3’UTR full length and truncates (up panel). **c** & **d** The efficiency of BCRC-3 pull-down assay was verified by RT-PCR (**c**) or real-time PCR (**d**). GAPDH was used as negative control. Relative level of BCRC-3 was normalized to input. **e** qRT-PCR analysis of the expression of 7 candidates in the EJ and T24 T lysates after biotin-BCRC-3 pull-down assay. **f** RNA-FISH detection of BCRC-3 in T24 T cells. Nuclei were stained blue with DAPI. BCRC-3 was stained red with cy3. Locked nucleic acid miR-182-5p probes were labeled with Dig (scale bar, 10 μm). **g** qRT-PCR analysis of the expression levels of BCRC-3 or GAPDH in the EJ and T24 T lysates after biotin-miR-182-5p pull-down assay. GAPDH was used as negative control. **h** The luciferase activities of wild type p27 3’UTR and mutant p27 3’UTR after transfection with miR-182-5p mimic in EJ or T24 T cell lines. **i** & **j** qRT-PCR and western blot analysis of the expression levels of p27 in EJ or T24 T cells after co-transfection with BCRC-3 overexpression vector and miR-182-5p mimics. (Data are mean ± SEM of three experiments. Student’s *t-*test analyzed the difference in **a**-**b**, **d**-**e**, **g**-**i**. * *P* < 0.01 vs. vector, p27 3’UTR, oligo probe, biotin-miR-182-5p WT, or mimic NC. & *P* < 0.05 vs. mimic NC. # *P* < 0.05 vs. mimic NC + BCRC-3)

To determine how BCRC-3 regulated p27 mRNA expression, we constructed the p27 3’-UTR and promoter luciferase reporters. The results indicated that ectopic expression of BCRC-3 significantly enhanced p27 3’-UTR activity in EJ and T24 T cells, while p27 promoter activity was not affected (Fig. 4a). To further confirm the exact locus, we divided the whole region of p27 3’UTR into three parts and each sequence was structured into the plasmid (pMIR-REPORT) separately (Fig. 4b). We called them T1, T2 and T3. Luciferase reporter assays indicated that T3 (1126–1344), but not T1 (1–800) or T2 (801–1125) contained the essential regulatory elements. Considering that miRNAs have been recognized as important regulators on mRNA 3’UTR, we used the miRanda database to analyze potential miRNA binding sites in 1126-1344 bp region of p27 3’UTR. Seven potential binding miRNA were selected, including miR-495-3p, miR-24-3p, miR-377-3p, miR-96-5p, miR-182-5p, miR-1271-5p and miR-194-5p. Next, we used RNA pull-down assay to identify whether BCRC-3 could directly bind these candidate miRNAs. The biotin-labeled probe was proved to pull down BCRC-3 and the pull-down efficiency was significantly increased upon BCRC-3 overexpression (Fig. 4c and d). It showed that miR-182-5p was the only one that abundantly pulled down by BCRC-3 in EJ and T24 T cells (Fig. 4e). Consistently, the merged images (yellow) obtained from FISH detection indicated that BCRC-3 and miR-182-5p were co-localized in BC cells (Fig. 4f). In addition, the data in RNAhybrid suggest that BCRC-3 contain three predictive binding sites of miR-182-5p (ΔG < − 18 kcal/mol) (Additional file 2: Figure S4a). We next transfected biotinylated miR-182-5p or its mutant (Additional file 2: Figure S4b) into EJ or T24 T cells stably transfected with BCRC-3 to further confirm the direct binding of miR-182-5p and BCRC-3. As shown in Fig. 4g, a significantly higher enrichment of BCRC-3 were captured in wild type biotin-miR-182-5p transfected cells compared with its mutant type.

To investigate the function of miR-182-5p in BC cell lines, we performed the miRNA inhibition experiments. Transfection of anti-miR-182-5p inhibitor obviously suppressed cell growth in EJ and T24 T cells (Additional file 2: Figure S3c-e). Real-time PCR and western blot assays demonstrated that anti-miR-182-5p inhibitor could increase the expression of p27 (Additional file 2: Figure S3a & b). In addition, we constructed wild type and mutant of p27 3’UTR luciferase reporter (Additional file 2: Figure S4c) to further explore the role of miR-182-5p in regulation of p27 3’UTR activity. The results indicated that miR-182-5p overexpression could significantly impair p27 3’UTR luciferase reporter activity, but there was no significant effect on the mutant of p27 3’UTR luciferase reporter activity (Fig. 4h), suggesting that miR-182-5p was able to directly bind to p27 3’UTR and block its activity. Consistently, ectopic expression of miR-182-5p could significantly decrease the expression of p27 on both mRNA and protein levels (Fig. 4i and j). Moreover, overexpression of BCRC-3 partly reversed the effects that miR-182-5p caused (Fig. 4i and j). The verification of BCRC-3 overexpression in these co-transfected cells was provided in Additional file 2: Figure S2c. The above results demonstrated that BCRC-3 could directly bind to miR-182-5p in BC cells, thereby promoting the expression of the downstream effector p27.

### MJ suppresses cell proliferation through targeting BCRC-3/miR-182-5p/p27 axis in BC cells

**Fig. 5:**
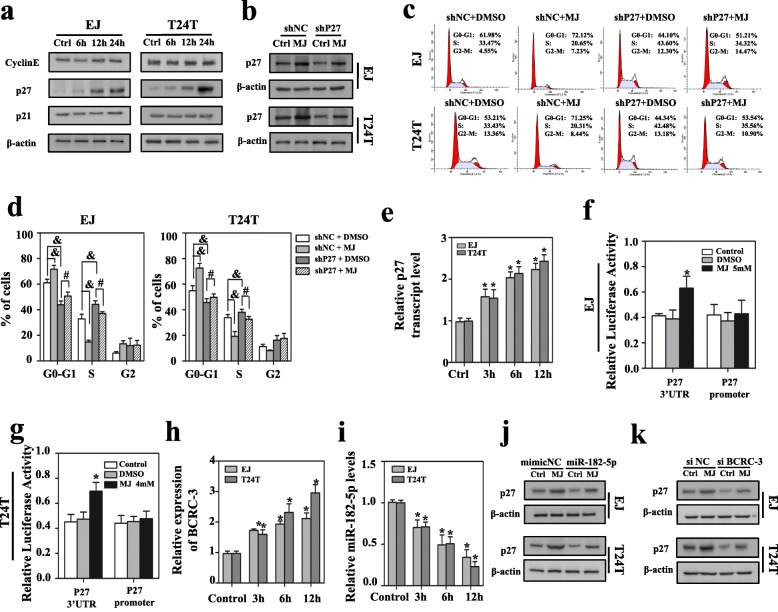
MJ inhibits cell proliferation through BCRC-3/miR-182-5p/p27 axis in BC cells. **a** Western blot analysis of the expression levels of cell cycle related proteins (p27, CyclinE, p21) in EJ or T24 T cells at different time points after MJ treatment. **b** Western blot analysis of the expression levels of p27 after MJ treatment in cells that were stably transfected with shP27 cells. **c** & **d** Cell cycle distributions in p27 knockdown cells after MJ treatment were presented by flow cytometry. **e** qRT-PCR analysis of the expression levels of p27 in EJ or T24 T cells after MJ treatment for different time points. **f** & **g** The luciferase activities of p27 3’UTR and promoter in EJ or T24 T cells after MJ treatment for 24 h. **h** & **i** qRT-PCR analysis of the expression levels of BCRC-3 (**h**) and miR-182-5p (**i**) in EJ or T24 T cells after MJ treatment for different time points. **j** & **k** Western blot analysis of the expression levels of p27 after MJ treatment in EJ or T24 T cells transfected with miR-182-5p mimics or si BCRC-3. (The data indicated mean ± SEM of three experiments. Student’s *t*-test analyzed the difference in **d**-**i**. & *P* < 0.05 vs. shNC + DMSO. # *P* < 0.05 vs. shP27 + DMSO. * *P* < 0.01 vs. control)

Our previous studies had shown that Methyl jasmonate had inhibitory effect on the growth of BC cells [28]. In this study, we further found that MJ exposure resulted in an increase of p27 expression at protein level (Fig. 5a), which was effectively abolished by knockdown of p27 (Fig. 5b). The cell cycle assay showed that treatment with MJ induced cell cycle arrest at G0/G1 phase in EJ and T24 T cells compared with vehicle control. Meanwhile, knockdown of p27 presented the opposite effects. Importantly, the inhibitory effects of MJ on cell cycle arrest were partly reversed in p27 knockdown cells (Fig. 5c and d). These findings suggested that p27 was involved in MJ-inhibition of BC cell proliferation.

**Fig. 6:**
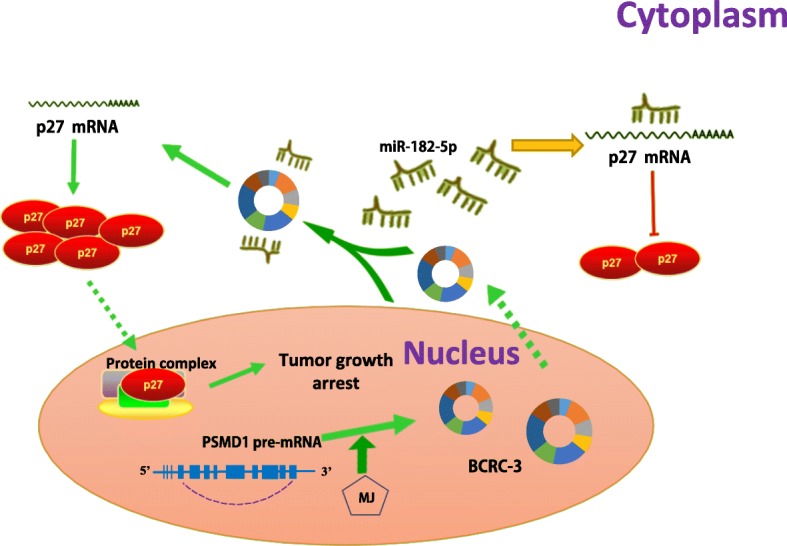
Schematic diagram indicates the BCRC-3 regulating pathway in BC cells

To figure out how MJ promoted the expression of p27, we detected p27 mRNA levels in EJ and T24 T cells at different time points after MJ treatment. As shown in Fig. 5e, MJ could induce a time-dependent up-regulation of p27 mRNA expression in BC cells. Dual-luciferase reporter assay showed that p27 3’UTR activity was obviously promoted in EJ and T24 T cells after MJ treatment, whereas DMSO (vehicle control) treatment only slightly changed p27 3’UTR or promoter activity (Fig. 5f and g). These data suggested that MJ could increase p27 mRNA expression by targeting its 3’UTR. Importantly, MJ treatment caused a time-dependent up-regulation of BCRC-3 expression in EJ and T24 T cells (Fig. 5h). Consistently, the expression of miR-182-5p was negatively regulated when treated with MJ (Fig. 5i). Moreover, transfection of miR-182-5p mimics partly abolished MJ-induced increase of p27 expression (Fig. 5j). On the other hand, knockdown of BCRC-3 (Additional file 2: Figure S2d) could partly reverse the promotion of p27 by MJ (Fig. 5k). Taken together, these data indicate that MJ could regulate the expression of p27 through BCRC-3/miRNA-182-5p/p27 axis (Fig. 6).

## Discussion

During the past years, hundreds of circRNAs have been reported to function as important drivers of tumorigenesis or tumor suppressors in distinct human cancers [29]. One important function pattern for circRNAs is acting as inhibitors of miRNAs by direct binding. CircRNAs which were regarded as miRNA sponge have some characteristics in common. Firstly, they derive from one or more exons of known protein-coding genes through back-splicing [30]. Second, subcellular location of these circRNAs in cell lines is predominantly in the cytoplasm, which occupies the same space of miRNA [31]. Finally, circRNAs with more predicting putative miRNA binding sites are likey to play the role of ceRNA for miRNA [21]. Our High-Throughout Sequencing had confirmed 524 down-regulated circRNAs and 47 up-regulated circRNAs in BC tissues [22]. Subsequently, we focused on the down-regulated circRNAs first, and in this study, we identified BCRC-3 which consists of nine exons (1,002 bp) from the PSMD1 gene, mainly locating in the cytoplasm. Our miRNA-targeting analysis showed that BCRC-3 harbors 3 targeting sites for miR-182-5p, suggesting that BCRC-3 may act as a miRNA sponge. RNA FISH demonstrated that BCRC-3 and miR-182-5p are co-localized in cells. Therefore, our results provided further evidence to support the notion that circRNAs can regulate gene expression via acting as “miRNA sponge”. It is suggested that exogenous expression of lowly expressed circRNAs can be performed by gene therapy where DNA cassettes designed for circRNA expression are delivered [32]. On the other hand, it is worthy to explore the roles of the up-regulated circRNAs to find novel therapeutic targets for the clinical application of BC.

To date only a few circRNAs with multiple binding sites for a single miRNA have been discovered and many circRNAs may have other roles in regulating cellular function. Like other noncoding RNAs, some circRNAs could function as protein decoys. With regard to this aspect, circFoxo3 harbors binding sites for p21 and CDK2. As a result, this ternary complex blocked cell cycle progression [33].Another interesting example is circPABPN1, which is derived from the PABPN1 gene [34]. The competitive binding of circPABPN1 to HuR prevents HuR binding to PABPN1 mRNA and reduces its translation. Unlike most circRNAs, circular intronic RNAs (ciRNA) and EIciRNAs are usually retained in the nucleus, where they may regulate transcription or alternative splicing [35]. Apart from the functions mentioned above, some endogenous circRNAs with an IRES or open reading frames have potential abilities to translate proteins with the help of modifications [36, 37], which challenges the conventional concept of non-coding RNAs. Therefore, apart from miRNA sponge, other functions of BCRC-3 still need to be elucidated in BC cells.

P27 (CDKN1B) is firstly identified as a negative regulator that halts cycle progression at G1/S phase. Abundant interacting proteins of p27 have been identified in recent years, indicating new roles for p27 in several CDK-unrelated processes [38, 39]. It is verified that the function of p27 protein is mainly regulated by post-translational modifications, especially phosphorylation of particular amino acid [40, 41], which alter the cellular localization and the degradation. However, the expression of p27 protein could also be regulated by transcriptional and posttranscriptional mechanisms. Accordingly, human tumors with an abnormal p27 metabolism/localization show poor prognosis and decreasing median survival time. Our recent studies demonstrated that ectopic expression of miR-182-5p blocked p27 3’UTR activity, whereas mutation of the binding sites at p27 3’UTR effectively reversed this inhibition [42]. Our data presented here indicated that BCRC-3 could interact with miR-182-5p to promote the expression of p27. Hence, we provided further evidence for the posttranscriptional regulation of p27 by circular RNA in cancer cells.

Among the naturally occurring jasmonates and their synthetic derivatives, Methyl jasmonate (MJ) showed the highest activity in accordance with cytotoxicity and induction of apoptosis in human cancer cells [43–45]. Besides, increasing evidence indicated that MJ could suppress the proliferation of urological malignancy [28, 46] depending on cellular mRNA transcription and protein translation [47, 48]. We have reported that MJ possessed high selectivity anticancer function toward BC cells by inducing apoptosis [28]. In the present study, we explored the effects of MJ on BC cell cycle progression and demonstrated that MJ could promote p27 expression through BCRC-3/miRNA-182-5p/p27 axis. Nevertheless, the mechanism underlying MJ-induced BCRC-3 expression still needs to be investigated in future studies.

## Conclusions

In summary, we firstly demonstrate that BCRC-3 is down-regulated in BC tissues and cell lines for the first time. BCRC-3 is capable of functioning as ceRNA for miR-182-5p to regulate the expression of p27. Moreover, our results show that cytotoxic MJ boosts the p27 protein via increasing the BCRC-3 expression, thus inhibiting the proliferation of BC cells. Our results not only explain the potential mechanisms related to circRNA in regulation BC cell proliferation, but they also make circRNA as a promising therapeutic target for BC treatment.

## Additional files

Additional file 1: **Table S1.** Clinicopathological features of 47 BC patients and the expression of BCRC-3 and miR-182-5p. **Table S2** Detailed information of primers and RNA sequences used in this study. (ZIP 22 kb)

Additional file 2: **Figure S1.** (**a**) qRT-PCR analysis of the transfection efficiency of si BCRC-3 vectors in BC cells. (**b-d**) Flow cytometry, EdU assay and cloning formation assay indicated the effect of BCRC3 KD on cell growth. (**e-f**) qRT-PCR and western blot analysis of the expression levels of p27 in the cells with KD of BCRC-3. (Data are mean ± SEM of three experiments. Student’s *t*-test analyzed the difference in **a-d**, **f**. * P<0.01 vs. vector). **Figure.S2** (**a**) qRT-PCR and western blot analysis of the expression levels of p27 after transfected with four p27 shRNAs in BC cells. (**b**) qRT-PCR assay indicating the expression of BCRC-3 in co-transfected cells (Fig. 3f & 3g). (**c**) qRT-PCR analysis of the expression of BCRC-3 in BC cells after co-transfection (Fig. 4i & 4j). (**d**) qRT-PCR assay indicating the expression of BCRC-3 after MJ treatment in the cells with KD of BCRC-3 (Fig. 5k). (Data are mean ± SEM of three experiments. Student’s *t*-test analyzed the difference in** a-d**. * P<0.01 vs. shNC, vector + shNC, or vector + shP27. & P<0.05 vs. mimic NC or siNC + control. # P<0.05 vs. miR-182-5p or siBCRC-3 + control). **Figure.S3** (**a-b**) qRT-PCR and western blot analysis of the expression levels of p27 in cells with KD of miR-182-5p. (**c-e**) Flow cytometry, EdU assay and cloning formation assay indicated the effect of the inactivation of miR-182-5p on cell growth. (Data are mean ± SEM of three experiments. Student’s *t*-test compared the difference in **b-e**. * P<0.01 vs. anti-NC). **Figure.S4** (**a**) The bioinformatics program RNAhybrid showed the detailed information of three binding sites of miR-182-5p on BCRC-3. (**b**) Biotin-coupled miR-182-5p wild-type and mutant sequences. (**c**) Schematic Sequence of the intact miR-182-5p-binding site in wide-type (WT) p27 mRNA 3’-UTR and its mutation (Mut) of p27 3’UTR luciferase reporter. (ZIP 1185 kb)

## Abbreviations

BC: Bladder cancer
BCRC-3: Bladder cancer related circRNA-3
CDK2: Cyclin-dependent kinase 2
CDK4: Cyclin-dependent kinase 4
CDK6: Cyclin-dependent kinase 6
ceRNAs: Competing endogenous RNAs
CircRNAs: Circular RNAs
EdU: 5-Ethynyl-20-deoxyuridine
FISH: Fluorescence in situ hybridization
MJ: Methyl jasmonate
MREs: MiRNA response elements
ncRNAs: Noncoding RNAs
p21: Cyclin-dependent kinase inhibitor 1A
p27: Cyclin-dependent kinase inhibitor 1B
qRT-PCR: Quantitative real-time PCR

## References

[1] Siegel RL, Miller KD, Jemal A (2017). Cancer statistics, 2017. CA Cancer J Clin.

[2] Leal J, Luengo-Fernandez R, Sullivan R, Witjes JA (2016). Economic burden of bladder Cancer across the European Union. Eur Urol.

[3] Burger M, Grossman HB, Droller M, Schmidbauer J, Hermann G, Dragoescu O, Ray E, Fradet Y, Karl A, Burgues JP (2013). Photodynamic diagnosis of non-muscle-invasive bladder cancer with hexaminolevulinate cystoscopy: a meta-analysis of detection and recurrence based on raw data. Eur Urol.

[4] Kamat AM, Hahn NM, Efstathiou JA, Lerner SP, Malmström P-U, Choi W, Guo CC, Lotan Y, Kassouf W (2016). Bladder cancer. Lancet.

[5] Babjuk M, Bohle A, Burger M, Capoun O, Cohen D, Comperat EM, Hernandez V, Kaasinen E, Palou J, Roupret M (2017). EAU guidelines on non-muscle-invasive urothelial carcinoma of the bladder: update 2016. Eur Urol.

[6] Stenzl A, Cowan NC, De Santis M, Jakse G, Kuczyk MA, Merseburger AS, Ribal MJ, Sherif A, Witjes JA (2009). The updated EAU guidelines on muscle-invasive and metastatic bladder cancer. Eur Urol.

[7] Cambier S, Sylvester RJ, Collette L, Gontero P, Brausi MA, van Andel G, Kirkels WJ, Silva FC, Oosterlinck W, Prescott S, et al. EORTC nomograms and risk groups for predicting recurrence, progression, and disease-specific and overall survival in non-muscle-invasive stage ta-T1 urothelial bladder Cancer patients treated with 1-3 years of maintenance Bacillus Calmette-Guerin. Eur Urol. 2016;69:60-9. 10.1016/j.eururo.2015.06.045 26210894

[8] Knollman Hayley, Godwin J. Luke, Jain Rishi, Wong Yu-Ning, Plimack Elizabeth R., Geynisman Daniel M. (2015). Muscle-invasive urothelial bladder cancer: an update on systemic therapy. Therapeutic Advances in Urology.

[9] Jeck WR, Sharpless NE (2014). Detecting and characterizing circular RNAs. Nat Biotechnol.

[10] Wilusz JE, Sharp PA (2013). Molecular biology. A circuitous route to noncoding RNA. Science.

[11] Hsu MT, Coca-Prados M (1979). Electron microscopic evidence for the circular form of RNA in the cytoplasm of eukaryotic cells. Nature.

[12] Sanger HL, Klotz G, Riesner D, Gross HJ, Kleinschmidt AK (1976). Viroids are single-stranded covalently closed circular RNA molecules existing as highly base-paired rod-like structures. Proc Natl Acad Sci U S A.

[13] Salzman J, Chen RE, Olsen MN, Wang PL, Brown PO (2013). Cell-type specific features of circular RNA expression. PLoS Genet.

[14] Jeck WR, Sorrentino JA, Wang K, Slevin MK, Burd CE, Liu J, Marzluff WF, Sharpless NE (2013). Circular RNAs are abundant, conserved, and associated with ALU repeats. RNA.

[15] Rybak-Wolf A, Stottmeister C, Glazar P, Jens M, Pino N, Giusti S, Hanan M, Behm M, Bartok O, Ashwal-Fluss R (2015). Circular RNAs in the mammalian brain are highly abundant, conserved, and dynamically expressed. Mol Cell.

[16] Hansen TB, Jensen TI, Clausen BH, Bramsen JB, Finsen B, Damgaard CK, Kjems J (2013). Natural RNA circles function as efficient microRNA sponges. Nature.

[17] Holdt LM, Stahringer A, Sass K, Pichler G, Kulak NA, Wilfert W, Kohlmaier A, Herbst A, Northoff BH, Nicolaou A (2016). Circular non-coding RNA ANRIL modulates ribosomal RNA maturation and atherosclerosis in humans. Nat Commun.

[18] Zheng Q, Bao C, Guo W, Li S, Chen J, Chen B, Luo Y, Lyu D, Li Y, Shi G (2016). Circular RNA profiling reveals an abundant circHIPK3 that regulates cell growth by sponging multiple miRNAs. Nat Commun.

[19] Memczak S, Jens M, Elefsinioti A, Torti F, Krueger J, Rybak A, Maier L, Mackowiak SD, Gregersen LH, Munschauer M (2013). Circular RNAs are a large class of animal RNAs with regulatory potency. Nature.

[20] Yu CY, Li TC, Wu YY, Yeh CH, Chiang W, Chuang CY, Kuo HC (2017). The circular RNA circBIRC6 participates in the molecular circuitry controlling human pluripotency. Nat Commun.

[21] Han D, Li J, Wang H, Su X, Hou J, Gu Y, Qian C, Lin Y, Liu X, Huang M (2017). Circular RNA circMTO1 acts as the sponge of microRNA-9 to suppress hepatocellular carcinoma progression. Hepatology.

[22] Li Y, Zheng F, Xiao X, Xie F, Tao D, Huang C, Liu D, Wang M, Wang L, Zeng F, Jiang G (2017). CircHIPK3 sponges miR-558 to suppress heparanase expression in bladder cancer cells. EMBO Rep.

[23] Li B, Xie F, Zheng FX, Jiang GS, Zeng FQ, Xiao XY (2017). Overexpression of CircRNA BCRC4 regulates cell apoptosis and MicroRNA-101/EZH2 signaling in bladder cancer. J Huazhong Univ Sci Technolog Med Sci.

[24] Luo G, Liu D, Huang C, Wang M, Xiao X, Zeng F, Wang L, Jiang G (2017). LncRNA GAS5 inhibits cellular proliferation by targeting P27(Kip1). Mol Cancer Res.

[25] Wu X, Liu D, Tao D, Xiang W, Xiao X, Wang M, Wang L, Luo G, Li Y, Zeng F, Jiang G (2016). BRD4 regulates EZH2 transcription through upregulation of C-MYC and represents a novel therapeutic target in bladder Cancer. Mol Cancer Ther.

[26] Huang H, Pan X, Jin H, Li Y, Zhang L, Yang C, Liu P, Liu Y, Chen L, Li J (2015). PHLPP2 downregulation contributes to lung carcinogenesis following B[a]P/B[a]PDE exposure. Clin Cancer Res.

[27] Huang H, Jin H, Zhao H, Wang J, Li X, Yan H, Wang S, Guo X, Xue L, Li J (2017). RhoGDIbeta promotes Sp1/MMP-2 expression and bladder cancer invasion through perturbing miR-200c-targeted JNK2 protein translation. Mol Oncol.

[28] Wang Y, Xiang W, Wang M, Huang T, Xiao X, Wang L, Tao D, Dong L, Zeng F, Jiang G (2014). Methyl jasmonate sensitizes human bladder cancer cells to gambogic acid-induced apoptosis through down-regulation of EZH2 expression by miR-101. Br J Pharmacol.

[29] Hanahan D, Weinberg RA (2011). Hallmarks of cancer: the next generation. Cell.

[30] Yang C, Yuan W, Yang X, Li P, Wang J, Han J, Tao J, Li P, Yang H, Lv Q, Zhang W (2018). Circular RNA circ-ITCH inhibits bladder cancer progression by sponging miR-17/miR-224 and regulating p21, PTEN expression. Mol Cancer.

[31] Zeng K, Chen X, Xu M, Liu X, Hu X, Xu T, Sun H, Pan Y, He B, Wang S (2018). CircHIPK3 promotes colorectal cancer growth and metastasis by sponging miR-7. Cell Death Dis.

[32] Kristensen L, Hansen T, Venø M, Kjems J (2018). Circular RNAs in cancer: opportunities and challenges in the field. Oncogene.

[33] Du WW, Yang W, Liu E, Yang Z, Dhaliwal P, Yang BB (2016). Foxo3 circular RNA retards cell cycle progression via forming ternary complexes with p21 and CDK2. Nucleic Acids Res.

[34] Abdelmohsen K, Panda AC, Munk R, Grammatikakis I, Dudekula DB, De S, Kim J, Noh JH, Kim KM, Martindale JL, Gorospe M (2017). Identification of HuR target circular RNAs uncovers suppression of PABPN1 translation by CircPABPN1. RNA Biol.

[35] Li Z, Huang C, Bao C, Chen L, Lin M, Wang X, Zhong G, Yu B, Hu W, Dai L (2015). Exon-intron circular RNAs regulate transcription in the nucleus. Nat Struct Mol Biol.

[36] Legnini I, Di Timoteo G, Rossi F, Morlando M, Briganti F, Sthandier O, Fatica A, Santini T, Andronache A, Wade M (2017). Circ-ZNF609 is a circular RNA that can be translated and functions in Myogenesis. Mol Cell.

[37] Yang Yibing, Gao Xinya, Zhang Maolei, Yan Sheng, Sun Chengjun, Xiao Feizhe, Huang Nunu, Yang Xuesong, Zhao Kun, Zhou Huangkai, Huang Suyun, Xie Bo, Zhang Nu (2017). Novel Role of FBXW7 Circular RNA in Repressing Glioma Tumorigenesis. JNCI: Journal of the National Cancer Institute.

[38] Hsieh HY, Shen CH, Lin RI, Feng YM, Huang SY, Wang YH, Wu SF, Hsu CD, Chan MW (2016). Cyproheptadine exhibits antitumor activity in urothelial carcinoma cells by targeting GSK3beta to suppress mTOR and beta-catenin signaling pathways. Cancer Lett.

[39] Zhao D, Besser AH, Wander SA, Sun J, Zhou W, Wang B, Ince T, Durante MA, Guo W, Mills G (2015). Cytoplasmic p27 promotes epithelial-mesenchymal transition and tumor metastasis via STAT3-mediated Twist1 upregulation. Oncogene.

[40] Molina-Sanchez P, Del Campo L, Esteban V, Rius C, Chevre R, Fuster JJ, Ferrer M, Redondo JM, Andres V (2018). Defective p27 phosphorylation at serine 10 affects vascular reactivity and increases abdominal aortic aneurysm development via Cox-2 activation. J Mol Cell Cardiol.

[41] Lai TY, Yen CJ, Tsai HW, Yang YS, Hong WF, Chiang CW (2016). The B56 gamma 3 regulatory subunit-containing protein phosphatase 2A outcompetes Akt to regulate p27KIP1 subcellular localization by selectively dephosphorylating phospho-Thr157 of p27KIP1. Oncotarget.

[42] Jiang G, Huang C, Li J, Huang H, Wang J, Li Y, Xie F, Jin H, Zhu J, Huang C (2018). Transcriptional and post-transcriptional upregulation of p27 mediates growth inhibition of isorhapontigenin (ISO) on human bladder cancer cells. Carcinogenesis.

[43] Flescher E (2007). Jasmonates in cancer therapy. Cancer Lett.

[44] Cohen S, Flescher E (2009). Methyl jasmonate: a plant stress hormone as an anti-cancer drug. Phytochemistry.

[45] Tong QS, Jiang GS, Zheng LD, Tang ST, Cai JB, Liu Y, Zeng FQ, Dong JH (2008). Natural jasmonates of different structures suppress the growth of human neuroblastoma cell line SH-SY5Y and its mechanisms. Acta Pharmacol Sin.

[46] Jiang G, Zhao J, Xiao X, Tao D, Gu C, Tong Q, Luo B, Wang L, Zeng F (2011). AN N-terminal Smac peptide sensitizes human prostate carcinoma cells to methyl jasmonate-induced apoptosis. Cancer Lett.

[47] Fingrut O, Flescher E (2002). Plant stress hormones suppress the proliferation and induce apoptosis in human cancer cells. Leukemia.

[48] Rotem R, Fingrut O, Moskovitz J, Flescher E (2003). The anticancer agent methyl jasmonate induces activation of stress-regulated c-Jun N-terminal kinase and p38 protein kinase in human lymphoid cells. Leukemia.

