# Supplementary material for: Circular RNA BCRC-3 suppresses bladder cancer proliferation through miR-182-5p/p27 axis
Source: Mol Cancer. 2018 Oct 3;17:144. doi: 10.1186/s12943-018-0892-z (PMC6169039; doi:10.1186/s12943-018-0892-z)
Supplement: Supplementary file 1 — Table S1. Clinicopathological features of 47 BC patients and the expression of BCRC-3 and miR-182-5p. Table S2 Detailed information of primers and RNA sequences used in this study. (ZIP 22 kb) [file 12943_2018_892_MOESM1_ESM.zip › Table.S1.docx]

**Table 1 Correlation between BCRC-3 or miR-182-5p expression and clinicopathological factors in bladder cancer**

| Parameters | Group | Cases | BCRC-3 expression | | | | P value | miR-182-5p expression | | | | P value |
| --- | --- | --- | --- | --- | --- | --- | --- | --- | --- | --- | --- | --- |
|  |  |  | Low | % | High | % |  | Low | % | High | % |  |
| Gendar | Male | 32 | 28 | 88 | 4 | 12 | 0.6638 | 6 | 19 | 26 | 81 | 0.7042 |
|  | Female | 15 | 12 | 80 | 3 | 20 |  | 4 | 27 | 11 | 73 |  |
| Age(years) | <55 | 25 | 18 | 72 | 7 | 28 | 0.7471 | 6 | 24 | 19 | 76 | 1.000 |
|  | ≥55 | 22 | 17 | 77 | 5 | 23 |  | 5 | 23 | 17 | 77 |  |
| Tumor stage | pTa-T1 | 18 | 12 | 67 | 6 | 33 | 0.7590 | 4 | 22 | 14 | 73 | 0.7441 |
|  | pT2-T4 | 29 | 17 | 59 | 12 | 41 |  | 8 | 28 | 21 | 72 |  |
| Tumor size | <3.0 cm | 16 | 7 | 44 | 9 | 56 | 0.3551 | 3 | 19 | 13 | 81 | 0.6758 |
|  | ≥3.0cm | 31 | 19 | 61 | 12 | 39 |  | 4 | 13 | 27 | 87 |  |
| Grade | Low | 23 | 11 | 48 | 12 | 52 | 0.2443 | 6 | 26 | 17 | 74 | 0.1365 |
|  | High | 24 | 16 | 67 | 8 | 33 |  | 2 | 8 | 22 | 92 |  |
| Lymph node metastasis | Absent | 30 | 14 | 47 | 16 | 53 | 0.5460 | 7 | 23 | 23 | 77 | 0.7334 |
|  | Present | 17 | 6 | 35 | 11 | 65 |  | 5 | 30 | 12 | 70 |  |

P < 0.05 represents statistical significance (Chi-square test).
