# Supplementary material for: Circular RNA BCRC-3 suppresses bladder cancer proliferation through miR-182-5p/p27 axis
Source: Mol Cancer. 2018 Oct 3;17:144. doi: 10.1186/s12943-018-0892-z (PMC6169039; doi:10.1186/s12943-018-0892-z)
Supplement: Supplementary file 2 — Figure S1. (a) qRT-PCR analysis of the transfection efficiency of si BCRC-3 vectors in BC cells. (b-d) Flow cytometry, EdU assay and cloning formation assay indicated the effect of BCRC3 KD on cell growth. (e-f) qRT-PCR and western blot analysis of the expression levels of p27 in the cells with KD of BCRC-3. (Data are mean ± SEM of three experiments. Student’s t-test analyzed the difference in a-d, f. * P<0.01 vs. vector). Figure.S2 (a) qRT-PCR and western blot analysis of the expression levels of p27 after transfected with four p27 shRNAs in BC cells. (b) qRT-PCR assay indicating the expression of BCRC-3 in co-transfected cells (Fig. 3f & 3g). (c) qRT-PCR analysis of the expression of BCRC-3 in BC cells after co-transfection (Fig. 4i & 4j). (d) qRT-PCR assay indicating the expression of BCRC-3 after MJ treatment in the cells with KD of BCRC-3 (Fig. 5k). (Data are mean ± SEM of three experiments. Student’s t-test analyzed the difference in a-d. * P<0.01 vs. shNC, vector + shNC, or vector + shP27. & P<0.05 vs. mimic NC or siNC + control. # P<0.05 vs. miR-182-5p or siBCRC-3 + control). Figure.S3 (a-b) qRT-PCR and western blot analysis of the expression levels of p27 in cells with KD of miR-182-5p. (c-e) Flow cytometry, EdU assay and cloning formation assay indicated the effect of the inactivation of miR-182-5p on cell growth. (Data are mean ± SEM of three experiments. Student’s t-test compared the difference in b-e. * P<0.01 vs. anti-NC). Figure.S4 (a) The bioinformatics program RNAhybrid showed the detailed information of three binding sites of miR-182-5p on BCRC-3. (b) Biotin-coupled miR-182-5p wild-type and mutant sequences. (c) Schematic Sequence of the intact miR-182-5p-binding site in wide-type (WT) p27 mRNA 3’-UTR and its mutation (Mut) of p27 3’UTR luciferase reporter. (ZIP 1185 kb) [file 12943_2018_892_MOESM2_ESM.zip › Figure S4.pdf]

Fig. S4

a

Target: BCRC-3 Length 1002bp

miRNA : miR-182-5p Length 24bp

mfe: -22.8 kcal/mol

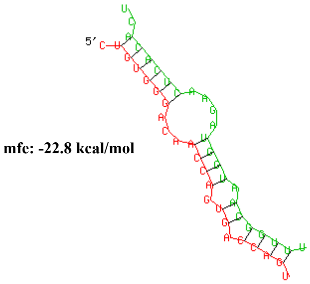

Binding site 1

mfe: -20.0 kcal/mol

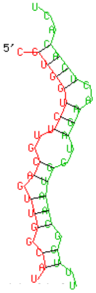

Binding site 2

mfe: -22.8 kcal/mol

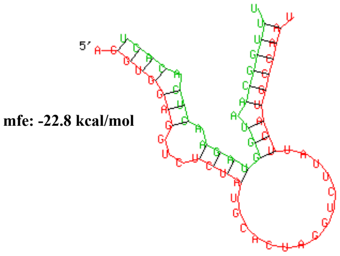

Binding site 3

b

Biotin-miR182-5p-Wild type
5’-UUUGGCAAUGGUAGAACUCACACU-3’

Biotin-miR182-5p-Mutant
5’-UAACCGCCUGGUAGAACUCACACU-3’

c

Position 1274-1296 of p27 3’UTR

WT 1274:5’…guaaugugugaaaaagaUGCCAAu 3’
| | | | | |
miR-182-5p:3’ucacacucaagaugguaACGGUUu 5’
| | x x x |
Mut 1274:5’…guaaugugugaaaaagaUGAUCAu
3’
